# Reply: In search of a better saphenous vein graft

**DOI:** 10.1016/j.xjon.2022.04.045

**Published:** 2022-05-04

**Authors:** Giovanni Jr Soletti, Mario Gaudino

**Affiliations:** Department of Cardiothoracic Surgery, Weill Cornell Medicine, New York, NY

Reply to the Editor:

**Figure:**
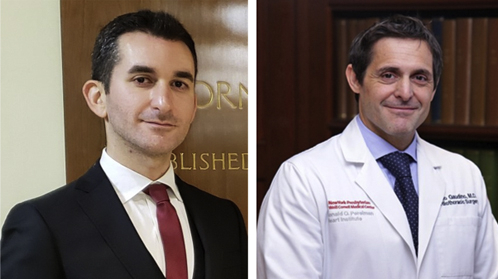
Giovanni Jr Soletti, MD, and Mario Gaudino, MD, PhD, MSCE

The authors reported no conflicts of interest.

The *Journal* policy requires editors and reviewers to disclose conflicts of interest and to decline handling or reviewing manuscripts for which they may have a conflict of interest. The editors and reviewers of this article have no conflicts of interest.

In a recent randomized trial reporting on the effect of external stenting on saphenous vein grafts (SVG) disease, Taggart and colleagues^1^ demonstrated that this technique improved Fitzgibbon patency scales and significantly reduced intimal hyperplasia area and thickness after a 2-year follow-up, although no difference in overall patency rates was observed between the stented and nonstented SVG (78.3% vs 82.2%, *P* = .43).^1^

In their letter, Lobo Filho and colleagues^2^ challenge the utility of external stenting, providing valid objections to its widespread use, such as uncertainties in long-term effectiveness and cost limitations as well as graft damage due to contact inflammatory response. The authors aim for a more systematic use of composite grafts that are more physiologic, economically sustainable, and may mitigate SVG pathologic remodeling. They report a small angiographic series of 14 patients who received an SVG to the right coronary system and an internal thoracic artery/saphenous vein composite graft to the left where aorta-anastomosed SVG have a significantly lower diameter compared with composite grafts (*P* < .001) after a mean follow-up of approximately 7 years.

While we agree in principle that composite grafting may have physiologic advantages compared with aorta-anastomosed grafting, the composite technique is more challenging and time-consuming, even in experienced hands, and is open to the risk of flow steal and diversion.^3^

Despite solid evidence and clear recommendations, the adoption of arterial grafting remains slow in current coronary artery bypass surgery practice, even in ideal candidates.^4^ Despite its relatively high failure rate,^5^^,^^6^ the SVG is still (by far) the most commonly used bypass conduit.

Strategies to improve vein patency rates are therefore crucial to obtain better clinical outcomes and are very much welcomed. Both external stenting and composite grafts have their own Achilles’ heel, but both should be part of the armamentarium of the modern coronary artery bypass surgery specialist until better evidence will be available.
